# Understanding the olfactory role in post-COVID cognitive and neuropsychiatric manifestations

**DOI:** 10.3389/fpsyg.2024.1407887

**Published:** 2024-05-27

**Authors:** N Azcue, Rocio Del Pino, Olatz Saenz de Argandoña, Amaia Ortiz de Echevarría, Marian Acera, T. Fernández-Valle, N. Ayo-Mentxakatorre, Jose Vicente Lafuente, Marta Ruiz-Lopez, A. López de Munain, Inigo Gabilondo, J. C. Gómez-Esteban, B. Tijero-Merino

**Affiliations:** ^1^Neurodegenerative Diseases Group, Biobizkaia Health Research Institute, Barakaldo, Spain; ^2^Department of Neurology, Cruces University Hospital-OSAKIDETZA, Barakaldo, Spain; ^3^Department of Neurosciences, University of the Basque Country UPV/EHU, Leioa, Spain; ^4^Department of Neurology, Donostia University Hospital-OSAKIDETZA, San Sebastián, Spain; ^5^Department of Neurosciences, Biodonostia Health Research Institute, San Sebastián, Spain; ^6^Department of Neurosciences, University of the Basque Country UPV-EHU, San Sebastián, Spain; ^7^CIBERNED-CIBER, Institute Carlos III, Madrid, Spain; ^8^The Basque Foundation for Science, IKERBASQUE, Bilbao, Spain

**Keywords:** cognition, neuropsychiatry, neuropsychology, olfaction, post-covid condition

## Abstract

**Introduction:**

Olfactory dysfunction (OD) is frequent after SARS-CoV-2 infection. The aim of this study was to examine if long-term OD is common in post-COVID condition, and the relationship between olfaction, cognition, neuropsychiatric symptoms, and disease duration in these patients.

**Methods:**

This study included 121 participants with post-COVID condition and 51 healthy controls (HC). A comprehensive neuropsychological and neuropsychiatric assessment was conducted, encompassing various domains, including general cognition, processing speed, verbal fluency, attention, verbal memory, visual memory, visuoconstructive ability, visuospatial ability, abstraction, executive functions, anxious-depressive symptoms, general health perception, fatigue level, sleep quality, and olfaction. Statistical analyses were carried out to understand the relationship of OD with cognition, and its role as moderator variable.

**Results:**

In total, 25% of the post-covid patients had a reduced smell capacity, while only 9.3% of HC presented OD. Post-COVID patients had statistically significantly worse cognitive performance and clinical status than HC. Verbal fluency (AUC = 0.85, *p* < 0.001), and attention (AUC = 0.82, *p* < 0.001) were the variables that best discriminate between groups. OD seemed to be a moderator between fatigue and cognition, and between disease duration and attention (β = −0.04; *p* = 0.014).

**Discussion:**

The study highlights marked cognitive and neuropsychiatric sequelae in individuals post-COVID relative to HC. Olfactory impairment exhibits correlations with both cognitive performance and general health. Olfaction emerges as a potential prognostic marker owing to its moderating influence on disease severity indicators.

## Introduction

The post-COVID condition is a syndrome marked by persistent fatigue, shortness of breath, and “brain fog,” which can be developed after SARS-CoV-2 infection. This term, proposed by NICE, refers to those patients with symptoms that last at least 2 months and usually begin within 3 months after the infection [ICD-11 (RA02)] (Soriano et al., 2021). Approximately 10–15% of individuals recovering from COVID-19 develop this syndrome (Nalbandian et al., 2023). Autonomic dysfunction, headaches, myalgia, anxiety, and depression are prevalent symptoms among these patients (Nalbandian et al., 2023).

In addition to the mentioned symptoms, several studies have highlighted olfactory dysfunction (OD) in the patients with post-COVID condition. OD is a common manifestation of SARS-CoV-2 infection, affecting approximately 50% of individuals during the acute phase (Saniasiaya et al., 2021). This OD is typically sudden and temporary, indicating primarily peripheral inflammation (Kay, 2022). However, in roughly 10% of patients, OD persists for months (Araújo et al., 2021; Ciofalo et al., 2022), suggesting the loss of olfactory epithelium, a disruption in central olfactory processing networks, or a persistent inflammation in either the olfactory epithelium, the olfactory bulb, or both (Xydakis et al., 2021; Doty, 2022).

Olfactory dysfunction (OD) stands as an independent marker distinct from COVID-19 severity and respiratory involvement (Cristillo et al., 2021). Recent inquiries have associated the acute loss of smell during the initial phase with compromised overall cognition (Llana et al., 2023). Cognitive impairments after the infection, particularly affecting memory and executive functions, are evident in less than one-fifth of individuals with prolonged OD (Muccioli et al., 2023). Previous assessments have explored the correlation between cognition and olfaction in post-COVID condition, revealing a link between prolonged hyposmia and cognitive decline (Cristillo et al., 2021; Azcue et al., 2022). Furthermore, patients experiencing post-COVID conditions report heightened instances of “brain fog,” headaches, and semantic memory deficits correlating with the severity of OD (Di Stadio et al., 2022; Fiorentino et al., 2022). Long-term olfactory dysfunction has been linked with structural alterations in the central nervous system (Yus et al., 2022; Campabadal et al., 2023). Essentially, enduring OD following COVID-19 appears to result in damage to olfactory and limbic brain regions, which are interconnected with adjacent areas implicated in memory and attention (Xydakis et al., 2021; Yus et al., 2022; Muccioli et al., 2023), potentially contributing to observed cognitive deficits. This pattern of damage mirrors neurological degeneration observed in conditions such as Alzheimer’s disease, Parkinson’s disease, and Lewy body dementia (Kay, 2022).

While numerous studies underscore significant cognitive differences between patients with hyposmia/anosmia and those without OD, many lack an objective smell evaluation or fail to compare it with healthy controls (HC).

Therefore, this study aims to ascertain if long-term OD is a frequent sign in post-COVID condition, and its relationship with cognition, neuropsychiatric symptoms, and disease duration in this pathology.

## Methods

### Participants

This study included 121 participants with post-COVID condition and 51 HC, recruited trough the Neurology Department of the Cruces University Hospital. The sample of patients was completed with people who reported post-covid symptoms, and who met the specific criteria of the study. Sex, years of education, disease duration, and smoking habit were recorded for all participants.

The inclusion criteria included participants between 18 and 85 years old, with a sufficient understanding and communication skills. Patients diagnosed with post-COVID condition met the criteria proposed by the NICE guidelines(Soriano et al., 2021), in which signs and symptoms that develop during or after the infection consistent with COVID-19 continued for more than 12 weeks and were not explained by an alternative diagnosis. For the diagnosis of acute COVID-19, the valid diagnostic methods were a positive nasal PCR, the detection of IgG and/or IgM antibodies against SARS-CoV-2 or a medical report supporting the diagnosis. Exclusion criteria included respiratory disease lasting 12 weeks after the infection, having been admitted to an intensive care unit and/or having had severe bilateral pneumonia or other severe disease manifestations requiring hospitalization, pregnancy or lactation, severe trauma, alcoholism, drug addiction, severe heart disease, radiological diagnosis of brain structural pathology, concomitant diseases that could influence the cognitive assessment or the olfactory sense (except smokers), as well as patients who have received some immunomodulatory treatments were excluded.

The study protocol was approved by the Basque Drug Research Ethics Committee [*Comité de Ética de la Investigación con medicamentos de Euskadi* (CEIm-E) (PI2020210)]. All participants gave written informed consent prior to their participation in the study, in accordance with the tenets of the Declaration of Helsinki.

### Cognitive and neuropsychiatric assessment

A comprehensive neuropsychological assessment was conducted, encompassing various domains such general cognition screening (Montreal Cognitive Assessment [MoCA]), attentional verbal and working memory (Digits from Wechsler Adult Intelligence Scale IV [WAIS-IV]), visual attention (Trail Making Test A [TMT A]), sustained attention (Touluose-Piéron Revised [TP-R]), alternating attention (Trail Making Test B [TMT B]) verbal fluency (animals and words starting with P), processing speed (Symbol Digit Modality Test [SDMT] and Salthouse Perceptual Comparison Test [SPCT]), cognitive flexibility (Modified Wisconsin Card Sorting Test [M-WCST]), verbal memory (Hopkins Verbal Learning Test- Revised [HVLT-R], visual memory (Brief Visuospatial Memory Test-Revised [BVMT-R]), visuoconstructive capacity (Taylor Complex Figure Test [TCF]), visual perception (Benton Judgment Of Line Orientation [JLO]), inhibitory capacity (Stroop Test), and abstraction (similarities from WAIS-IV).

Neuropsychiatric and clinical status were also assessed with questionnaires measuring general health (The 36-Item Short Form Health Survey [SF-36]), impact of fatigue or fatigue levels (Modified Fatigue Impact Scale [MFIS]), depressive symptoms (the Short Form of Geriatric Depression Scale [GDS]), anxiety symptoms (State–Trait Anxiety Inventory [STAI]), suicidal ideation (Columbia Suicide Severity Rating Scale [C-SSRS]), and sleep quality (Pittsburgh Sleep Quality Index [PSQI]).

To evaluate olfaction, the Brief Smell Identification Test (BSIT) validated to Hispanic and non-Hispanic white population was employed, consisting of 12 distinct odors. The results were adjusted for age and sex, yielding four possible categories: normal, deficient, relatively abnormal, and abnormal (Doty, 2001; Menon et al., 2013).

### Statistics

Statistical analyses were conducted using IBM SPSS Statistics for Windows, version 26.0 (IBM SPSS, Armonk, NY, United States). Prior to analysis, normality and homogeneity of the variables within each group were assessed. The U Mann–Whitney test was employed to examine differences in age and years of education between groups, while the Chi-square test was used to assess differences in sex and smoking habits.

Normalized z-score values were computed for each test, and these z-scores were utilized to construct composites for each cognitive domain. ANCOVA was employed to investigate differences in cognitive domains and BSIT scores between post-COVID and HC groups, with age, sex, years of education, and smoking habits serving as covariates. Pearson bivariate correlations were calculated to explore the relationship between cognitive domains and BSIT *z*-scores.

ROC curve analysis was performed to identify variables that most effectively discriminated between patients and HC. Stepwise linear regression analysis was undertaken to identify symptoms and variables that best explained cognitive performance in post-COVID patients. Finally, a simple moderation analysis was executed, utilizing the results of the BSIT as a bicategorical moderator with the PROCESS v4.2 macro for SPSS. The BSIT results were categorized as normal and pathological, with the latter encompassing those with deficient, relatively abnormal, and abnormal outcomes. Statistical significance was established at *p* < 0.05 (two-tailed).

## Results

### Participants

Age, sex, years of education, and smoking habits were recorded for all participants, with disease duration additionally documented for patients with post-COVID condition (Table 1).

**Table 1:** Clinical and demographic data.

	HC (*n* = 51) *M* (*SD*)	Post-COVID (*n* = 121) *M* (*SD*)	Statistics
Age, years	42.17 (9.93)	46.29 (9.64)	*U* = 2369.50*
Education, years	16.70 (2.75)	16.15 (3.30)	*U* = 3480.50
Female, *n* (%)	41 (81.4%)	92 (76.0%)	*χ*^2^ = 0.39
Smoking habit			*χ*^2^ = 14.33**
Non-smoker	30 (58.8%)	80 (66.1%)	
Smoker	10 (19.6%)	5 (4.1%)	
Former smoker	9 (17.6%)	34 (28.1%)	
Disease duration, months		15.75 (8.19)	
SF-36	96.80 (9.61)	48.42 (24.17)	*U* = 5854.50***
MFIS	10.47 (11.77)	64.49 (14.73)	*U* = 47.00***
PSQI	5.08 (2.98)	10.89 (4.61)	*U* = 935.50***
STAI-state	11.74 (10.28)	28.07 (14.03)	*U* = 1021.00***
STAI-trait	12.98 (9.39)	16.62 (13.09)	*U* = 2566.50
GDS	1.11 (1.53)	7.23 (3.79)	*U* = 364.00***
C-SSRS	0.00 (0.00)	0.28 (0.70)	*U* = 2269.50***

*p ≤ 0.05; **p ≤ 0.01; ***p ≤ 0.001.C-SSRS, Columbia Suicide Severity Rating Scale; GDS, Geriatric Depression Scale; HC, healthy controls; MFIS, Modified Fatigue Impact Scale; PSQI, Pittsburgh Sleep Quality Index; SF-36, The 36-Item Short Form Health Survey; STAI, State–Trait Anxiety Inventory.

Statistical analyses revealed a significant difference in age between groups (*U* = 2369.50, *p* = 0.016), with the HC group being younger. Gender distribution and years of education did not exhibit significant differences between the groups. However, there were notable distinctions in smoking habits (*χ*^2^ = 14.33, *p* = 0.006). In the post-COVID condition group, 66.1% were non-smokers, 4.1% were smokers, and 28.1% were former smokers. Conversely, in the HC group, 58.8% were non-smokers, 19.6% were smokers, and 17.6% were former smokers.

### Cognitive and neuropsychiatric status

**Figure 1:**
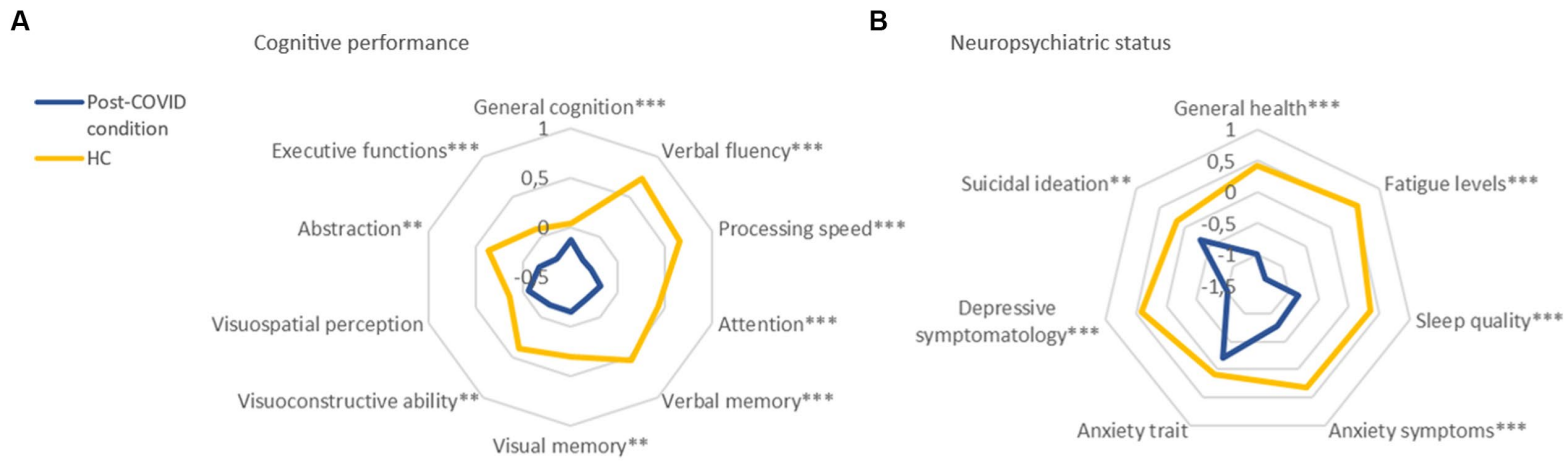
Cognitive performance and neuropsychiatric status in post-COVID condition and HC. **(A)** Cognitive performance. **(B)** Neuropsychiatric status. Values are shown in *z*-scores. A higher z indicates a better cognition or neuropsychiatric status. *p ≤ 0.05; **p ≤ 0.01; ***p ≤ 0.001. C-SSRS, Columbia Suicide Severity Rating Scale; GDS, Geriatric Depression Scale; HC, healthy controls; MFIS, Modified Fatigue Impact Scale; PSQI, Pittsburgh Sleep Quality Index; SF-36: The 36-Item Short Form Health Survey; STAI, State–Trait Anxiety Inventory.

Significant differences between groups were observed in multiple cognitive domains: general cognition (*F*_1,169_ = 35.59, *p* < 0.001), verbal fluency (*F*_1,169_ = 68.77, *p* < 0.001), processing speed (*F*_1,169_ = 48.14, *p* < 0.001), attention (*F*_1,169_ = 27.54 *p* < 0.001), verbal memory (*F*_1,169_ = 30.58, *p* < 0.001), visual memory (*F*_1,169_ = 6.74, *p* = 0.010), visuoconstructive ability (F_1,169_ = 7.31, *p* = 0.001), abstraction (F_1,169_ = 9.69, *p* = 0.002), and executive functions (*F*_1,169_ = 15.03, *p* = 0.008). Post-COVID condition patients exhibited significantly poorer performance in all cognitive domains, except for general cognition and visuospatial perception (Figure 1A).

Similarly, the neuropsychiatric status was examined, with comparisons between groups adjusted for age and sex. Statistically significant differences were found in general health (*F*_1,169_ = 314.72, *p* < 0.001), fatigue levels (*F*_1,169=_508.65, *p* < 0.001), sleep quality (*F*_1,169_ = 63.15, *p* < 0.001), anxiety symptoms (*F*_1,169_ = 51.03, *p* < 0.001), depressive symptomatology (*F*_1,169_ = 114.48, *p* < 0.001), and suicidal ideation (*F*_1,169_ = 6.73, *p* = 0.010). Post-COVID condition patients exhibited a clinically worse status compared to HC. However, no statistically significant differences between groups were observed in anxiety trait (Figure 1B).

**Figure 2:**
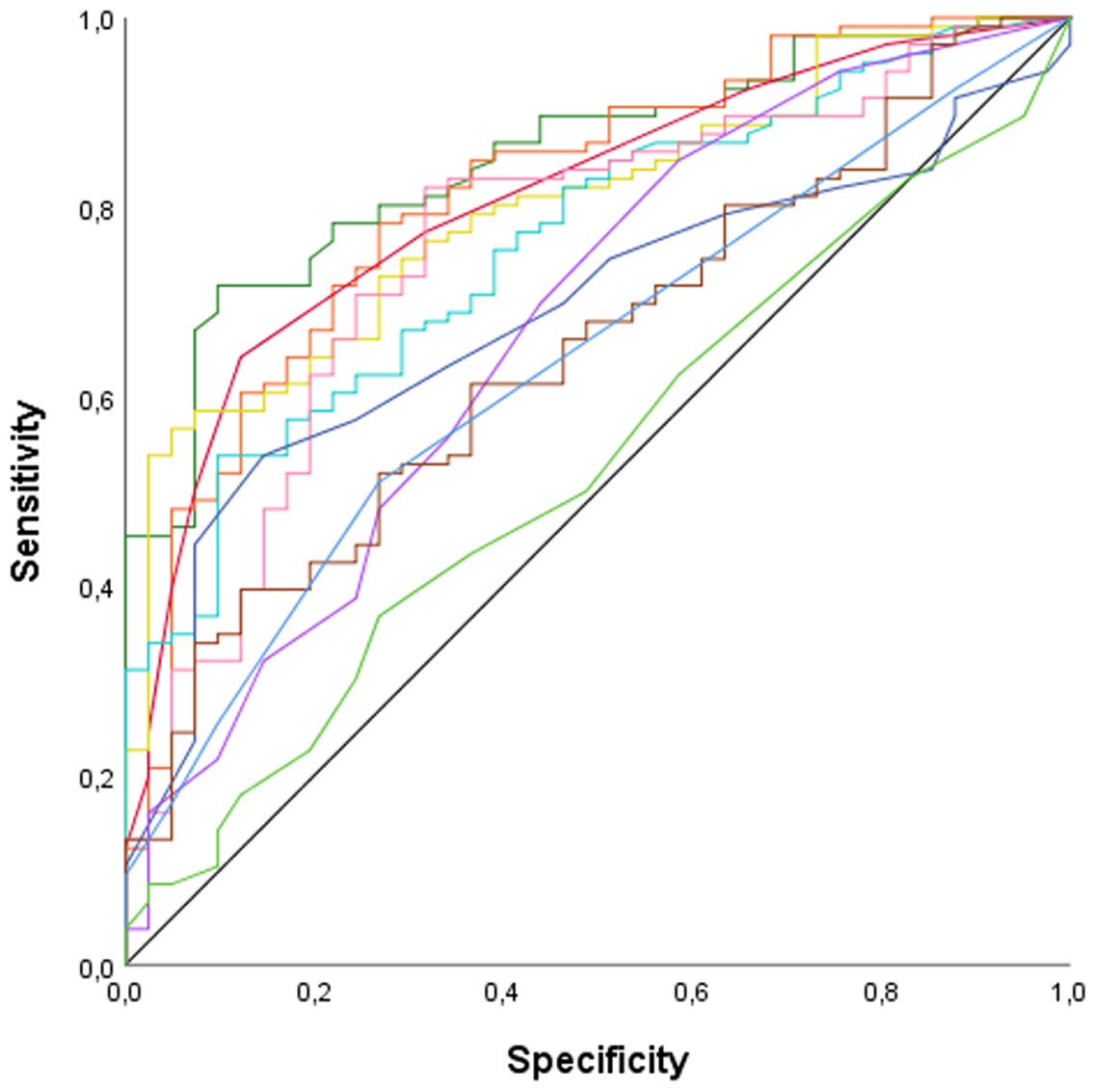
ROC curve. AUC, Area Under the Curve. **—**Abstraction (AUC = 0.68, *p* < 0.001); **—**Attention (AUC = 0.80, *p* < 0.001); **—**Executive functions (AUC = 0.76, *p* < 0.001); **—**General cognition (AUC = 0.81 *p* < 0.001); **—**Olfactory sense (AUC = 0.63, *p* = 0.011); **—**Processing speed (AUC = 0.81, *p* < 0.001); **—**Verbal fluency (AUC = 0.85, *p* < 0.001); **—**Verbal memory (AUC = 0.76, *p* < 0.001); **—**Visual memory (AUC = 0.65, *p* = 0.005); **—**Visuoconstructive ability (AUC = 0.67, *p* = 0.001); —Visuospatial perception (AUC = 0.53, *p* = 609).

ROC curve analysis was conducted to identify the domains that most effectively discriminated between patients and HC (Figure 2). The analysis revealed that verbal fluency (AUC = 0.85, *p* < 0.001), processing speed (AUC = 0.81, *p* < 0.001), general cognition (AUC = 0.81 *p* < 0.001), and attention (AUC = 0.80, *p* < 0.001), were the variables with the highest discriminatory capacity between patients and HC. Verbal memory (AUC = 0.76, *p* < 0.001), executive functions (AUC = 0.76, *p* < 0.001), abstraction (AUC = 0.68, *p* < 0.001), visuoconstructive ability (AUC = 0.67, *p* = 0.001), visual memory (AUC = 0.65, *p* = 0.005), and BSIT (AUC = 0.63, *p* = 0.011) also displayed discriminatory ability, albeit with lower AUC values. Visuospatial perception was the only cognitive domain that did not show discriminatory power between patients and HC.

Gender differences in individuals with post-COVID condition were examined, with age, years of education, and disease duration serving as covariates. The analysis revealed statistically significant differences in processing speed (*F*_1,118_ = 4.14, *p* = 0.044) and visuospatial perception (*F*_1,118_ = 15.52, *p* < 0.001), indicating that women exhibited poorer performance in these domains. However, no statistically significant differences were observed in general health, fatigue levels, sleep quality, anxiety-state, anxiety-trait, depressive symptoms, or suicidal ideation between men and women with post-COVID condition.

Regarding HC group, differences between gender were only found in visuospatial ability (*F*_1,49_ = 5.39, *p* = 0.025), with women having a better performance in this task.

### Olfaction

**Figure 3:**
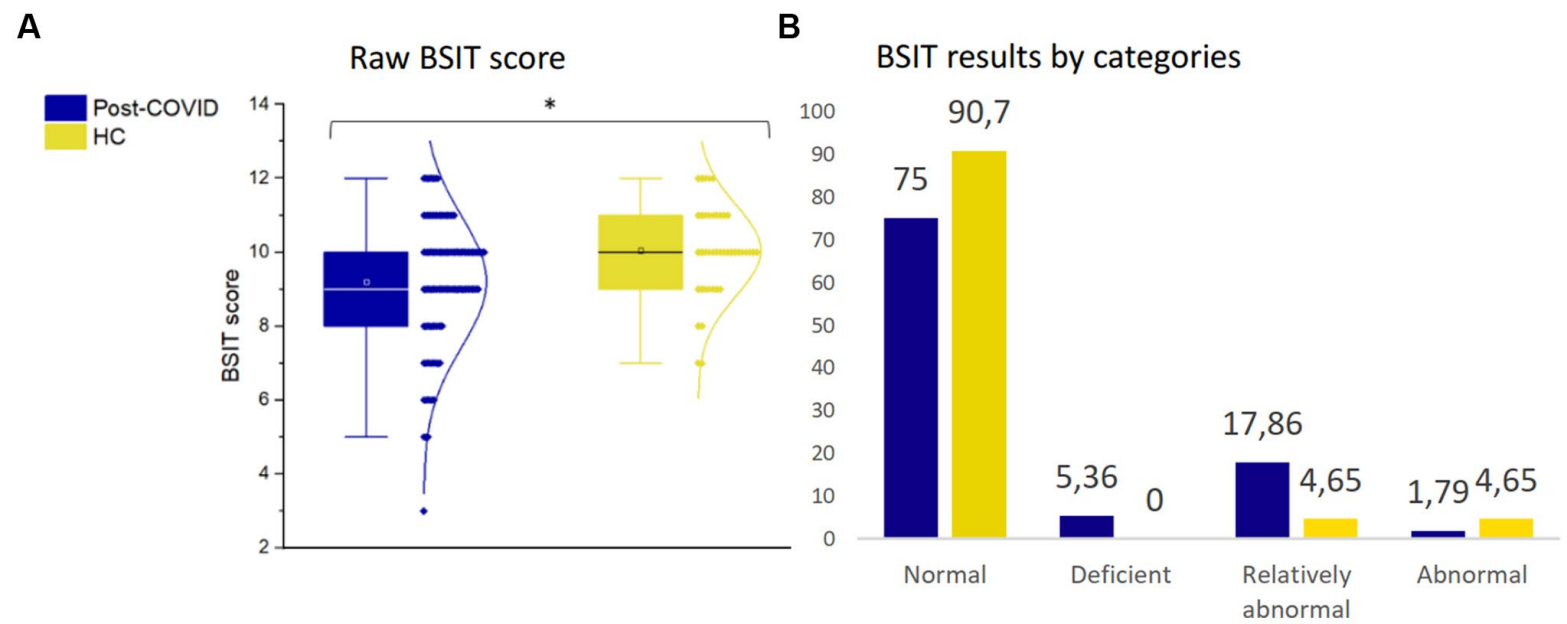
Olfactory assessment. **(A)** Raw BSIT score. **(B)** BSIT results by categories. *p ≤ 0.05. BSIT, Brief Smell Identification Test; HC, healthy controls.

Significant differences were identified between patients with post-COVID condition and HC (*F*_1,169_ = 4.94, *p* = 0.028), indicating that post-COVID patients exhibited lower olfactory performance (Figure 3A). Specifically, 25% of the patients displayed a reduced smell capacity (deficient, relatively abnormal or abnormal), whereas only 9.3% of the HC had OD (Figure 3B).

Furthermore, there were no statistically significant differences in olfaction between men and women among post-COVID patients. This analysis was adjusted for age, years of education, smoking habits, and disease duration. Additionally, no statistically significant differences in olfactory sense were observed among post-COVID patients based on disease duration.

The correlations between participants’ olfactory sense and their cognitive performance were examined. In post-COVID patients, olfactory sense correlated positively with attention (*r* = 0.23, *p = 0.*013), visuospatial perception (*r* = 0.26, *p* = 0.005), and abstraction capacity (*r* = 0.19, *p = 0.*049). Additionally, the olfaction was correlated with general health (*r* = 0.19, *p* = 0.044). These findings indicate that better olfactory sense was associated with improved cognitive performance and better general health in post-COVID patients. However, disease duration did not show a correlation with olfaction.

In HC, olfactory sense did not exhibit correlations with any cognitive domains or neuropsychiatric tests.

**Figure 4:**
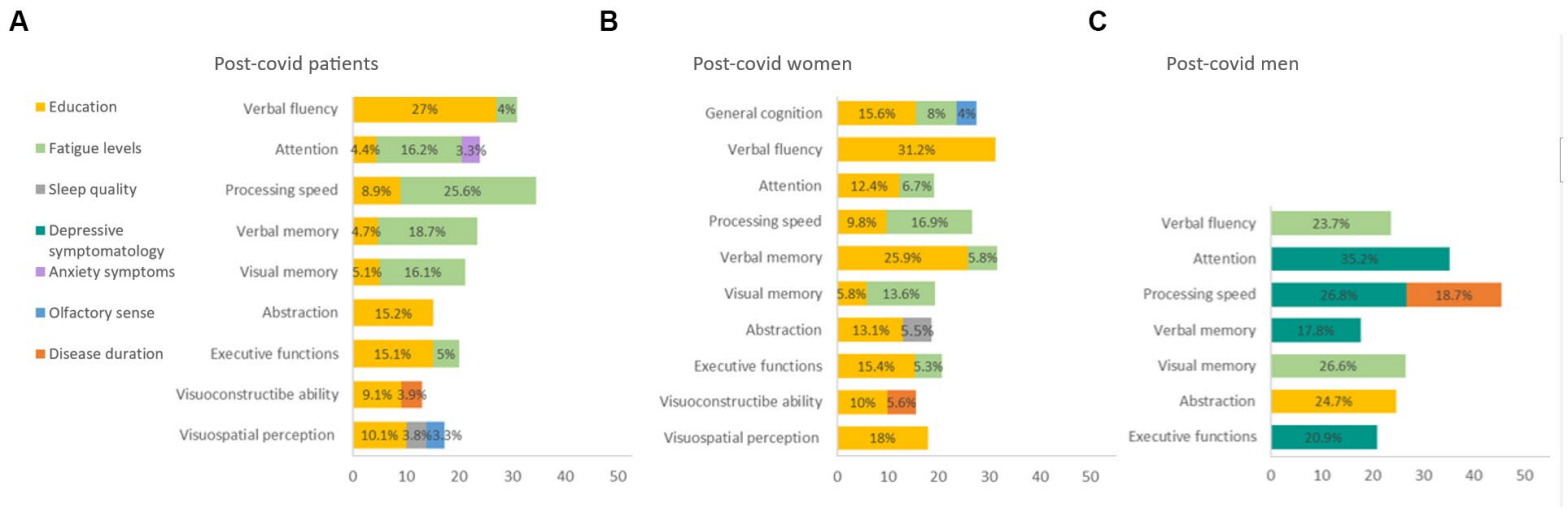
Linear regression of cognitive domain. **(A)** Post-covid patients. **(B)** Post-covid women. **(C)** Post-covid men. BSIT: Brief Smell Identification Test; MFIS: Modified Fatigue Impact Scale; PSQI: Pittsburgh Sleep Quality Index; STAI: State–Trait Anxiety Inventory.

A linear regression analysis of cognitive domains, symptomatology, BSIT, disease duration, and years of education was conducted. Fatigue, along with education level, were identified as the variables that best explained cognitive performance in post-COVID patients (4.4–27%). Olfactory ability explained 3.3% of visuospatial perception (Figure 4A). Additionally, when performing separate linear regressions for women and men with post-COVID condition, it was found that fatigue most influenced cognitive performance in women (5.3–16.9%) (Figure 4B), whereas in men, depressive symptoms played a more significant role (17.8–35.2%) (Figure 4C).

**Figure 5:**
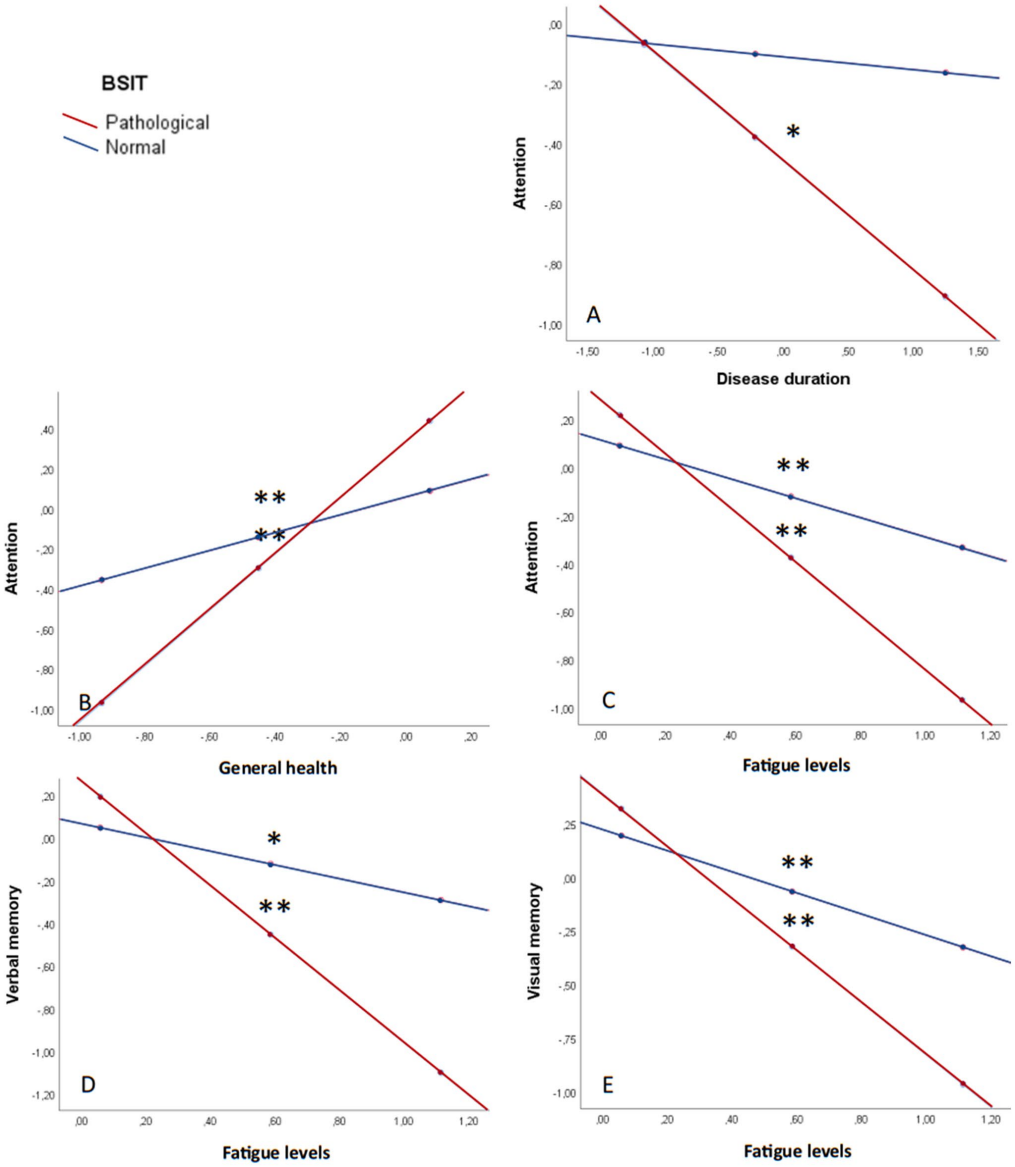
Olfaction as a moderating variable in cognitive performance. **(A)** Attention and disease duration. **(B)** Attention and general health. **(C)** Attention and fatigue. **(D)** Verbal memory and fatigue. **(E)** Visual memory and fatigue. All graphs are shown in *z* values. *p ≤ 0.05; **p ≤ 0.001; MFIS: Modified Fatigue Impact Scale; SF-36: The 36-Item Short Form Health Survey; STAI: State–Trait Anxiety Inventory.

Finally, simple moderation analyses were conducted with the olfactory sense (pathological/normal) serving as the moderating variable between neuropsychiatric status, clinical data, and cognitive performance (Figure 5). The results revealed differences in the interaction between disease duration and attention (β = −0.03; *p* = 0.051) (Figure 5A). Specifically, patients with a pathological olfaction demonstrated worse attention capacity as the duration of the disease increased (β = −0.04; *p* = 0.014).

Furthermore, general health had a statistically significant influence on attention, being moderated by olfaction capacity (β = −0.95; *p* = 0.003) (Figure 5B). Fatigue levels, as well, impacted attention (β = 0.72; *p* = 0.021) (Figure 5C), verbal memory (β = 0.90; *p* = 0.018) (Figure 5D), and visual memory (β = 0.72; *p* = 0.051) (Figure 5E), with worse performance the higher the fatigue and the worse olfaction capacity.

## Discussion

The objective of this study was to investigate whether long-term OD is a prominent feature of post-COVID condition and to explore the associations between olfaction, cognitive performance, and neuropsychiatric symptoms in these patients. Additionally, we aimed to present the neuropsychologic and neuropsychiatric profile of individuals with post-COVID condition.

Regarding olfaction, post-COVID patients exhibited a poorer sense of smell compared to HC. Our findings align with similar studies examining long-term hyposmia without post-COVID condition (Zhu et al., 2021). Acute-phase OD can result from various factors, including inflammation, while long-term OD may also be attributed to neurological alterations, in addition to persistent inflammation (Xydakis et al., 2021; Doty, 2022). In our sample, 25% of participants experienced long-term OD, a proportion similar to that reported in the general population following SARS-CoV-2 infection (Boscolo-Rizzo et al., 2022), but higher than what has been seen in other long-term studies (Araújo et al., 2021; Ciofalo et al., 2022), probably because of the higher proportion of women in our sample, who report smell dysfunction in approximately 2.5 more times than men (Araújo et al., 2021; Mendes Paranhos et al., 2022). The prevalence of OD in the general population is estimated to range from 3–22%, with upper airway infections, sinonasal diseases, and head trauma being the primary cause (Brämerson et al., 2004; Auinger et al., 2021; Renaud-Charest et al., 2021; Schäfer et al., 2021). In our sample, HC showed abnormal olfaction in 4.7%, and relatively abnormal in 4.7% of the cases.

Post-COVID patients displayed worse performance in various cognitive domains, general health, sleep quality, and reported higher levels of fatigue, anxiety symptoms, depressive symptomatology, and suicidal ideation compared to HC (Altuna et al., 2021; Ceban et al., 2022; Cavaco et al., 2023). Notably, these differences were more pronounced in female patients, consistent with previous studies (Ya’qoub et al., 2021; Sylvester et al., 2022). The prevalence of post-COVID condition also appears to be higher in women than men, and women tend to experience more fatigue and neurological symptoms in the post-COVID phase (Sylvester et al., 2022).

In examining the relationship between olfactory sense and cognitive performance, correlations were found with attention, visuospatial perception, and abstraction capacity in post-COVID patients. OD was also correlated with poorer general health. Importantly, these correlations were not observed in HC. This study reinforces the connection between damage to central nervous system areas related to smell and cognitive performance (Cristillo et al., 2021; Campabadal et al., 2023; Muccioli et al., 2023). While significant differences in olfactory ability were identified between patients and HC, fatigue levels emerged as the primary explanatory variable for cognitive performance in these patients. Specifically, fatigue levels explained a higher percentage of cognitive performance in women, whereas depressive symptoms played a more significant role in cognitive performance in men.

Olfaction emerged as a robust moderator variable, influencing the relationship between fatigue, memory, attention, and disease duration and attention capacity. These findings suggest a more pronounced cognitive decline in individuals with OD as the disease progresses. Although no differences in the proportion of patients with OD were observed between those with and without post-COVID condition, these results underscore the significance of long-term OD as an indicator of cognitive deterioration. This implies the need for comprehensive follow-up for post-COVID patients, indicating similarities between OD in post-COVID condition and other pathologies affecting cognitive performance and olfactory sense, such as Parkinson’s disease or Alzheimer’s disease (Rethinavel et al., 2021; Damiano et al., 2022; Di Stadio et al., 2022; Doty, 2022; Kay, 2022). In contrast, in HC, OD may be attributed to other factors like smoking habits (Vennemann et al., 2008). Some researchers propose that inflammatory stimuli causing OD and affecting the central nervous system could accelerate neurodegenerative processes (Xydakis et al., 2021). Impaired olfactory sense could be a prognostic factor, not only in elderly people, but also in different pathologies such as post-covid condition (Delgado-Lima et al., 2023).

Strengths of this study include the sample size of post-COVID patients, who underwent a thorough neuropsychologic and neuropsychiatric assessment, along with an objective olfaction evaluation. However, limitations include the lack of matching in age, gender, and years of education between the HC and patient groups, although statistical controls were applied to address these variables. The lack of information about the SARS-CoV-2 mutation with which the patients were infected was also a limitation due to its involvement in the olfactory sense.

In conclusion, individuals with post-COVID condition exhibited impaired olfactory ability, cognitive performance, and poorer mental health compared to HC. Interestingly, the proportion of post-COVID patients experiencing OD was comparable to that observed in individuals who had SARS-CoV-2 infection but did not develop post-COVID condition. Notably, OD emerged as a moderator, influencing the relationship between fatigue levels, disease duration, and cognitive performance in post-COVID patients. These findings underscore the multi-faceted impact of post-COVID condition on various aspects of health and highlight the potential role of OD as a significant factor in the cognitive well-being of affected individuals.

## Data availability statement

The datasets presented in this article are not readily available because anonymized data not published within this article will be made available upon reasonable request. Requests to access the datasets should be directed to RP, delpinorocio@gmail.com; JG-E, juancarlos.gomezesteban@gmail.com.

## Ethics statement

The studies involving humans were approved by Basque Drug Research Ethics Committee [Comité de Ética de la Investigación con medicamentos de Euskadi (CEIm-E) (PI2020210)]. The studies were conducted in accordance with the local legislation and institutional requirements. The participants provided their written informed consent to participate in this study.

## Author contributions

NA: Formal analysis, Investigation, Methodology, Writing – original draft, Writing – review & editing. RD: Conceptualization, Formal analysis, Investigation, Supervision, Validation, Writing – original draft, Writing – review & editing, Methodology. OS: Investigation, Writing – original draft, Writing – review & editing, Validation. AO: Investigation, Writing – original draft, Writing – review & editing. MA: Investigation, Writing – original draft, Writing – review & editing. TF-V: Investigation, Writing – original draft, Writing – review & editing. NA-M: Investigation, Writing – original draft, Writing – review & editing. JL: Conceptualization, Writing – original draft, Writing – review & editing. MR-L: Investigation, Writing – original draft, Writing – review & editing. AL: Writing – original draft, Writing – review & editing. IG: Writing – original draft, Writing – review & editing. JG-E: Writing – original draft, Writing – review & editing, Funding acquisition, Investigation, Methodology, Resources, Supervision. BT-M: Funding acquisition, Investigation, Methodology, Resources, Supervision, Writing – original draft, Writing – review & editing.
